# Occult Hepatitis B virus infection in previously screened, blood donors in Ile-Ife, Nigeria: implications for blood transfusion and stem cell transplantation

**DOI:** 10.1186/s12985-016-0533-3

**Published:** 2016-05-05

**Authors:** Amadin A. Olotu, Adesola O. Oyelese, Lateef Salawu, Rosemary A. Audu, Azuka P. Okwuraiwe, Aaron O. Aboderin

**Affiliations:** Department of Medical Microbiology and Parasitology, Bowen University/Bowen University Teaching Hospital, P.O. Box 15, Ogbomoso, Oyo State Nigeria; Department of Medical Microbiology and Parasitology, Obafemi Awolowo University Teaching Hospitals Complex, Ile-Ife, Nigeria; Department of Haematology and Blood Transfusion, Obafemi Awolowo University Teaching Hospitals Complex, Ile-Ife, Nigeria; Nigerian Institute for Medical Research, Lagos, Nigeria

**Keywords:** Occult hepatitis B Virus, Blood donors, HBV DNA, HBsAg, Anti-HBc

## Abstract

**Background:**

Hepatitis B virus (HBV) transmission through blood transfusion is reduced by screening for hepatitis B surface antigen (HBsAg). However this method cannot detect the presence of occult hepatitis B virus infection. This study sought to determine the prevalence of occult hepatitis B virus infection among blood donors in Ile-Ife, Nigeria. For the first time in Nigeria we employed an automated real-time PCR- method to investigate the prevalence of occult HBV in blood donors.

**Methods:**

Blood donors screened with HBsAg immunochromatographic rapid test kits at the blood transfusion units of two hospitals and found to be negative were recruited into the study. Questionnaires to elicit risk factors for HBV infection were administered and then 10 ml of blood was collected from each donor. Plasma samples obtained from these HBsAg negative blood donors were screened again for HBsAg using an enzyme-linked immunosorbent assay (ELISA) method, and those found negative were screened for the presence of total antibody to the HBV core antigen (anti-HBc) using ELISA method. Those positive to anti-HBc were then tested for HBV DNA, using an automated real-time PCR method.

**Results:**

Five hundred and seven blood donors found HBsAg negative by immunochromatographic rapid test kits at both blood transfusion units, were tested for HBsAg using ELISA and 5 (1 %) were HBsAg positive. The 502 found negative were tested for anti-HBc and 354 (70.5 %) were found positive implying previous exposure to HBV and 19 (5.4 %) of the 354 anti-HBc positive had HBV DNA signifying occult HBV infection. No risk factors were found to be associated with the presence of HBV DNA among those who tested positive.

**Conclusion:**

Occult HBV infection exists in blood donors in Ile-Ife, Nigeria and the use of HBsAg alone for screening prospective donors will not eliminate the risk of HBV transmission in blood transfusion or stem cell transplantation.

## Background

Hepatitis B virus (HBV) infection is a global, public health issue [1–3] of immense importance. It occurs worldwide and up to two billion people, approximately 30 % of the world’s population, have been infected globally [2]. Of this, 300–400 million people are chronically infected, approximating to about 5 % of the world’s population at risk of developing the complications of chronic HBV infection [1–5]. Deaths resulting from HBV yearly, stand at about 500,000 to 1.2 million worldwide. Most of these are due to sequelae of chronic HBV infection such as cirrhosis, liver failure and hepatocellular carcinoma (HCC) [2–5].

Blood transfusion could be an important route for the transmission of infection especially when donated blood is not screened for HBV infection [6]. Screening of donated blood for Hepatitis B surface Antigen (HBsAg) was introduced in the 1970s [7–10]. This greatly reduced HBV transmission due to blood transfusions [9, 11] as blood found to be HBsAg positive was not transfused.

In many developing countries including Nigeria, screening of blood donors or blood donated, for HBsAg alone, is still the only practice on which the prevention of HBV transmission during blood transfusion is based [12].

Numerous scientific papers have highlighted the presence of HBV infection in some individuals negative for HBsAg but having detectable HBV DNA in the liver or blood and some of these publications have documented HBV transmission resulting from transfusion of blood tested and found to be HBsAg negative [9, 13–15].

Occult HBV infection (OBI) has been the subject of numerous publications focusing on different aspects of and issues relating to OBI [10, 16–51]. It is a term that has been chosen by experts [33] to denote HBV infections in which HBsAg cannot be detected and the presence of infection is only established by amplifying and detecting HBV DNA [18–22, 28, 33, 34, 46].

One significant importance of OBI is the risk of transmission of HBV from individuals with OBI to recipients. This could occur if blood or blood components, stem cells or solid organs are transfused or transplanted following negative HBsAg results in donors with OBI.

Such infections could manifest overtly becoming HBsAg positive with possibly fatal consequences, the risk rising with immune suppressed recipients [14–16, 18, 25, 33, 46, 52, 53].

In Nigeria, which is highly endemic for HBV infection [54–57], current blood banking practices do not include procedures that would identify OBI and prevent transfusion of blood or blood products from apparently healthy donors with OBI to recipients [58].

As the prevalence of OBI tends to be higher where the prevalence of overt HBV infection is high [27] there may be a relatively high prevalence of OBI in Nigeria. All of these translating to an increasing number of patients who may be at risk of HBV infection from occult donors. Nevertheless there is limited data on the prevalence of OBI in blood donors in this country as only HBsAg screening is still relied upon [12, 58].

Against this background, we sought to investigate the prevalence of occult HBV in this region, to enable us make evidence-based recommendations for effective HBV screening to prevent HBV transmission from donors with OBI to recipients during blood transfusion.

## Methods

### Ethical issues

Approval was sought and obtained from the Ethics and Research Committee of Obafemi Awolowo University Teaching Hospitals Complex (OAUTHC) Ile-Ife with reference number IRB/IEC/0004553 and the Ethics and Research Committee of Seventh Day Adventist Hospital (SDAH), Ile-Ife. Prospective subjects were informed about the study and written consent was obtained from those who agreed to participate. The study was a prospective, cross-sectional study.

### Selection of study population

Apparently healthy blood donors who had been screened and found eligible by the respective blood banks for donation were recruited over a period of six and a half months, from June 2013 to January 2014. Subjects who had hepatitis B vaccination in the previous one month were excluded.

### Sociodemographic information

With the aid of a structured questionnaire, relevant sociodemographic information was obtained from blood donors. This included age, sex, number of lifetime sexual partners, presence of tatoos/scarification marks, history of alcohol use, smoking, sharing of sharps and hepatitis B vaccination. The questionnaires were self-administered by subjects or any subjects who needed help in filling the questionnaires were assisted by trained volunteers who were medical doctors, medical laboratory scientists, medical laboratory science students, and laboratory technologists.

### Collection of specimens

Ten milliliters (mls) of venous blood was collected from each consecutive consenting, eligible, previously screened, apparently healthy blood donor, donating to the blood banks at OAUTHC and SDAH in Ile-Ife. The blood was collected aseptically into a specimen bottle containing potassium ethylene diamine tetra acetate (K^+^EDTA) anticoagulant. All samples were centrifuged at room temperature at 3500 rpm for 10 min within 24 h of collection. The plasma was then separated and stored at −70 °C until analysed.

### Serological analysis

The serological studies were done at the department of Medical Microbiology and Parasitology, OAUTHC. HBsAg was tested for in all specimens using commercially available Monolisa™ HBsAg ULTRA ELISA kits manufactured by BIORAD (3,*bd Raymond Poincare, 92430 Marnes-la-Coquette-France*) with a lower limit of detection estimated to be less than 0.13 IU/ml, according to the manufacturer’s instructions.

All HBsAg negative plasma samples were then tested for total anti-HBc using commercially available Monolisa™ Anti-HBc PLUS ELISA kits by BIORAD (3,*bd Raymond Poincare, 92430 Marnes-la-Coquette-France*) according to the manufacturer’s instructions.

### HBV DNA studies

Detection and quantification of HBV DNA was done at the Human Virology Laboratory of the Nigerian Institute of Medical Research (NIMR) Lagos and all HBsAg negative, anti-HBc positive plasma samples were tested for HBV DNA using “COBAS AmpliPrep/COBAS TaqMan HBV Test version 2.0” kits made by Roche (Roche Molecular Systems Inc. Branchburg, NJ 08876 USA) using the “Cobas Ampliprep instrument and COBAS TaqMan 48 Analyzer” with a limit of detection of less than 20 IU/ml for HBV DNA. All procedures were performed according to manufacturer’s instructions.

Initial daily maintenance and priming were performed and specimens and controls were brought to room temperature. Reagent cassettes and disposables were then loaded onto the COBAS AmpliPrep Instrument as per manufacturer’s instructions. Sample racks were prepared, then 650 μl of High Positive control (HPC), Low Positive Control (LPC), Negative control (NC) and each specimen was transferred using a micropipette and DNase-free tips into the appropriate sample input tubes (S-tubes) The sample racks with S-tubes and K-tubes were then loaded onto the appropriate rack position on the COBAS AmpliPrep Instrument.

The COBAS AmpliPrep Instrument was then started using the AMPLILINK software and automated specimen processing was done. Each set of processed specimens in K-tubes, on K- carriers were then manually transferred to the COBAS TaqMan 48 Analyzer using the K- carrier transporter and the COBAS TaqMan 48 Analyzer run was then started for automated real-time PCR amplification and detection and quantification. At the end of each run the results were printed out. Positive (HPC and LPC) and negative controls were included in each run.

### Data analysis and statistical techniques

Descriptive statistics were calculated and reported for sociodemographic characteristics. Percentages were used to describe frequency analyses of categorical variables. Chi-squared test was used to compare categorical variables. A P value < 0.05 was considered to indicate statistical significance. Data processing and statistical analyses were performed using Epi Info 7 Software by CDC.

## Results

### Hepatitis B surface antigen testing in donors

Five hundred and seven blood donors tested at both OAUTHC and SDAH blood banks for HBsAg using immunochromatographic rapid test kits and found negative were tested for HBsAg using ELISA and five of them were found to be HBsAg positive as presented in Table 1.

**Table 1:** HBsAg Screening by ELISA

Blood Bank	OAUTHC	SDAH	Total
Number Screened	405^a^	102^a^	507
Number Positive	4	1	5

^a^Previously screened and found HBsAg negative by the immunochromatographic rapid test kits

*HBsAg* Hepatitis B surface antigen, *OAUTHC* Obafemi Awolowo University Teaching Hospitals Complex, *SDAH* Seventh Day Adventist Hospital

### Assessment of HBsAg negative donors for anti-HBc

The 502 donors found to be HBsAg negative using ELISA tests were tested for anti-HBc also using ELISA. Three hundred and fifty four (71 %) were positive giving an anti-HBc prevalence of 71 % among blood donors.

### Real time PCR assays for HBV DNA

Out of the 354 HBsAg negative, anti-HBc positive subjects whose specimens were tested, HBV DNA was detected in 19 (5.4 %) as shown in Table 2. The viral load for those with OBI ranged from 68 to < 20 IU/ml.

**Table 2:** Quantitative HBV DNA results for HBsAg negative, anti-HBc positive subjects with occult HBV

Viral Load	Number of Subjects	HBsAg	Anti-HBc
<20 IU/ml	16	Negative	Positive
21 IU/ml	1	Negative	Positive
64 IU/ml	1	Negative	Positive
68 IU/ml	1	Negative	Positive

*HBsAg* Hepatitis B surface antigen, *Anti-HBc* antibody to hepatitis B core antigen, *IU/ml* international units per milliliter

### Summary of laboratory tests and results

See Fig. 1 below.

**Fig. 1:**
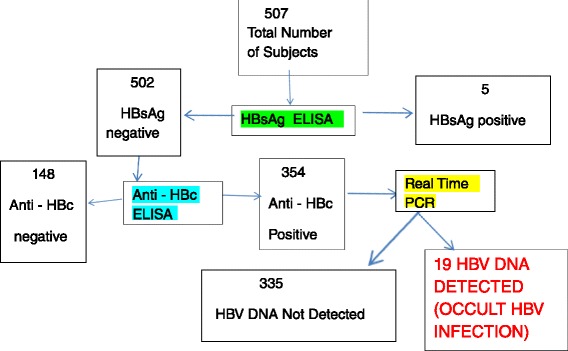
Summary of Results. Figure one summarizes the laboratory tests done and the results seen in form of a flow chart. There were 507 subjects and their plasma specimens were screened for HBsAg using ELISA and five were positive. The remaining 502 were then screened for anti-HBc also using ELISA and 354 were found to be positive. The 354 anti-HBc samples were then screened for HBV DNA using real time PCR. HBV DNA was found in 19. (HBV – Hepatitis B virus, HBsAg –Hepatitis B surface Antigen Anti-HBc – antibody to hepatitis B core antigen, ELISA – Enzyme linked immunosorbent assay, PCR – Polymerase chain reaction)

### Sociodemographic characteristics of subjects with occult HBV

Nineteen subjects were found to have occult HBV infection. All (100 %) of them were males and 17 (89.5 %) of them were less than 35 years (Table 3). There was no significant association between occult HBV infection and any of the variables tested as seen in Table 3.

**Table 3:** Sociodemographic characteristics of anti-HBc positive subjects with/without occult HBV infection

Variables	HBV DNA		Tests of Statistical Significance		
	positive(%)	negative(%)	Χ^2^	df	*p*
Age (years)			0.68	1	0.41
≤35 >35	17(89.5) 2(10.5)	270(82.1) 59(17.9)			
Sex			0.58	1	1.00
Male Female	19(100.0) 0(0.0)	325(97.0) 10(3.0)			
Alcohol			1.32	1	0.33
Yes No	10(52.6) 9(47.4)	131(39.3) 202(60.7)			
Smoking			1.98	1	0.25
Yes No	4(21.0) 15(79.0)	35(10.6) 295(89.4)			
Share Sharps			0.18	1	1.00
Yes No	2(14.3) 12(85.7)	50(18.9) 215(81.1)			
Scarification marks/Tattoos			0.17	1	0.78
Yes No	4(28.6) 10(71.4)	90(33.8) 176(66.2)			
Number of Lifetime Sexual Partners			0.21	1	0.78
≤1 >1	7(50.0) 7(50.0)	116(43.8) 149(56.2)			

*P.S* For the last three characteristics/variables no responses were available for five of those with occult HBV infection

*HBV* Hepatitis B virus

*Anti-HBc* antibody to hepatitis B core antigen, *X* ^*2*^ chi square, *df* degree of freedom, *p* p-value

## Discussion

In this study we found in HBsAg negative blood donors an anti-HBc prevalence of 70.5 %. This means over 70 % of our adult population have been infected with HBV at some point in their lives. This has previously been reported by others [59] including Kiire who in 1996 [60] reported that 72.5 % of Nigerians show evidence of exposure to HBV infection. This means that the burden of HBV infection has not changed significantly over the last 18 years especially in adults. This is not surprising as they were born before 2004 when hepatitis B vaccine actually became widely available as part of the universal immunization schedule for infants in Nigeria [61]. Other workers such as Japhet et al. [62] found a prevalence of 5.4 % for IgM anti-HBc only positive blood donors but did not look for total anti-HBc. Salawu et al. [63] also found about 4.4 % of anti-HBc in HBsAg negative donors but in that study rapid test kits were used which may be less sensitive than the ELISA used in this study.

This study found an OBI prevalence of 5.4 % among anti-HBc positive blood donors in Ile-Ife. This means that after HBsAg screening about 1 in 20 to 1 in 25 blood donations still have HBV DNA. Therefore the risk of transfusing blood from donors with OBI is about 1 in 20 donations. Published data on OBI in blood donors in Nigeria is sparse. Nna et al. [64] found a prevalence of 8 % in Abakaliki, South-Eastern Nigeria among 100 donors, Opaleye et al. [65] found a very high prevalence of 36 % among 429 donors while Oluyinka et al. [66] found a prevalence of 17 % in Southwestern Nigeria among 429 donors. These prevalence values are all higher than that found in this study. In all three, the method of HBV DNA detection was nested PCR. Nested PCR has the significant drawback of being prone to contamination and false positives especially during the sample transfer step preceding the second round of amplification [67–69] and so that may explain the much higher values. The problem of contamination and false positives in nucleic acid amplification assays is eliminated in automated real time PCR assays [70] such as the one we used in this study. However there may be differences in the prevalence of OBI among blood donors from one part of the country to another, reflecting differences in the prevalence of overt HBV infection which exist from one part of the country to the other [54–57].

Other studies on OBI in Nigeria have been in other subject populations such as Ola et al. [71] who found OBI in 2 of 28 chronic hepatitis patients in Ibadan, Opaleye et al. [72] also in Nigeria found a prevalence of 11.2 % of OBI in HIV positive patients using archived specimens from Ikole Ekiti, however this subjects were not likely to qualify as blood donors and as such no risk of their blood being transfused.

The prevalence in this study was higher than the 1.7 % found in Ghana by Zahn et al. [73] but this may be because the HBV DNA was tested for in all blood donors and not just those that were anti- HBc positive as was done in this study. HBsAg positive individuals who are anti-HBc positive have been found to be more likely to have OBI than those without anti-HBc.

In Brazil, Silva et al. found 3.3 % but used a PCR assay with a LLOD of about 200 IU/ml [23], which is less sensitive than the real-time PCR assay used in this study. This could have resulted in a lower detection of OBI.

The prevalence found in this study is much higher than what has been found in the US and some other western countries where only 0.1–2.4 % of HBsAg negative, anti-HBc positive blood donors were found to have HBV DNA [46, 74]. This is not surprising because only about 5 % of the population have come in contact with HBV in those regions unlike in Nigeria where over 70 % of the population have at some time in their lives been exposed to or infected with HBV [59, 60].

Manzini et al. in Italy [29] found 4.86 % among HBsAg negative, anti-HBc positive blood donors which is similar to what we found.

This prevalence is much smaller than the 38 % that was reported by Yotsuyanagi et al. in Japan [13] but the sample size was just 50 blood donors which is small compared to this study. This may also be the reason why Jafarzahdeh et al. found a 28.56 % prevalence in Iran [31] as they only assayed 14 HBsAg negative, anti-HBc positive samples for HBV DNA and found in four samples.

This study did not find any significant association between occult HBV and the variables tested such as age, sex, alcohol use, smoking, sharing of sharps scarification marks/tattoos and number of lifetime sexual partners. This is most likely because of the small number (19) of individuals in which we detected HBV DNA. This number was probably too small for reliable statistical analysis. However all those with OBI in this study were found to be males, mostly less than 36 years.

Of note is that in this study HBsAg was detected by ELISA in five (1 %) blood donors who had previously been screened with rapid test kits, declared HBsAg negative and cleared for donation. In at least two of these blood donors HBV DNA was demonstrated by real-time PCR. This shows that the rapid test kits being used for HBsAg screening in the blood banks where the subjects were recruited are not adequate for screening in blood transfusion services and would allow transfusion of HBsAg positive blood in 1 out of every 100 blood donations.

The inadequacy in sensitivity and variation in performance between different locations of some rapid test kits used in resource poor settings for HBsAg screening has been shown by Bjoerkvoll et al. [75]. In their cross-sectional epidemiological study they compared the accuracy of rapid test immunochromatographic kits in the detection of HBsAg, anti-HBc and anti-HCV against ELISA, in two populations of 1200 potential blood donors in rural Cambodia and Vietnam. For HBsAg specifically, they found the rapid test kits to be high in specificity (99.8–99.9 %) but lower in sensitivity (86.5 %). They also found a difference in its sensitivity between both countries. In Cambodia the sensitivity was 93.5 % and in Vietnam 81.8 %.

Anti-HBs prevalence has been studied in Nigeria. Salawu et. al [63] in a study in Ile-Ife among 457 blood donors negative for hepatitis B surface antigen(HBsAg) found a prevalence rate of 12.7 %, Japhet et. al [62] found a rate of 15.2 % among 92 donors also studied in Ile-Ife while Oluyinka et. al [66] found 35 % of those with OBI had anti-HBs. However we did not investigate the prevalence of anti-HBs among blood donors with or without OBI in our study because we did not have enough funds to do that.

## Conclusion

Hepatitis B virus infection is still endemic in Nigeria with a large percentage of the population showing evidence of prior exposure to HBV in this environment evidently from routes other than blood transfusion. There is a relatively high burden of occult HBV infection in our environment and the use of HBsAg alone for screening either in blood transfusion or transplantation services does not eliminate the risk of HBV transmission.

## Recommendations

We recommend that assays including rapid test kits used for HBsAg screening should be validated before routine use locally comparing their performance with at least 3^rd^ generation ELISA. Only ELISA assays or rapid test kits with comparable performance with regard to sensitivity or specificity should be used for HBsAg screening in blood transfusion.

Nucleic acid tests (NATs) should be introduced for routine screening of donors in blood transfusion and mandatorily for screening of donors and recipients in transplantation services. Individual donor (sample) testing using a NAT is more sensitive than testing in pools and so is preferred for this purpose [76–79].

## Limitations

Other HBsAg negative and anti-HBc negative specimens could not be tested by real time PCR because of the high costs involved. We also were not able to test for anti-HBs in the specimens of subjects with OBI because our funds were limited.
